# Supplementary material for: The Role of Velocity-Based Training (VBT) in Enhancing Athletic Performance in Trained Individuals: A Meta-Analysis of Controlled Trials
Source: Int J Environ Res Public Health. 2022 Jul 28;19(15):9252. doi: 10.3390/ijerph19159252 (PMC9368129; doi:10.3390/ijerph19159252)
Supplement: Supplementary file 1 [file ijerph-19-09252-s001.zip › ijerph-1795678-supplementary.pdf]

Study or Subgroup	Post Mean	Post SD	Post Total	Pre Mean	Pre SD	Pre Total	Weight	Std. Mean Difference IV, Random, 95% CI
**1.1.1 AL**								
Banyard et al. 2020 squat AL	153.6	19.4	12	136.7	18.9	12	4.5%	0.85 [0.01, 1.69]
Galiano et al. 2020 squat AL (a)	108.2	14.5	15	97.7	13.7	15	5.8%	0.72 [-0.02, 1.47]
Galiano et al. 2020 squat AL (b)	110.2	18.4	13	97	13.9	13	5.0%	0.78 [-0.02, 1.59]
Jiménez-Reyes et al. 2021 squat AL	96.39	16.18	13	85.52	16.47	13	5.1%	0.64 [-0.15, 1.44]
Pareja-Blanco et al. 2020 squat AL (a)	112.9	19.9	14	99.9	19.1	14	5.5%	0.65 [-0.12, 1.41]
Pareja-Blanco et al. 2020 squat AL (b)	113.6	15.6	14	96.7	15.3	14	5.0%	1.06 [0.26, 1.86]
Pareja-Blanco et al. 2020 squat AL (c)	110.9	19.6	13	97.8	23.2	13	5.1%	0.59 [-0.20, 1.38]
Pareja-Blanco et al. 2020 squat AL (d)	105.6	18.5	14	94.5	17.5	14	5.5%	0.60 [-0.16, 1.36]
Pareja - Blanco et al. 2017 squat AL (a)	125.2	12.3	12	106.5	12.2	12	3.8%	1.47 [0.55, 2.39]
Pareja - Blanco et al. 2017 squat AL (b)	118.6	20.4	10	104.5	15.1	10	3.8%	0.75 [-0.16, 1.67]
Riscart-López et al. 2021 squat AL (a)	110.7	15.7	11	94.4	17.1	11	4.0%	0.96 [0.06, 1.85]
Riscart-López et al. 2021 squat AL (b)	103.5	21.7	10	93.3	17.4	10	4.0%	0.50 [-0.40, 1.39]
Riscart-López et al. 2021 squat AL (c)	112	25.2	11	94.9	25.3	11	4.3%	0.65 [-0.21, 1.51]
Riscart-López et al. 2021 squat AL (d)	109.3	27.7	11	94.8	26.4	11	4.4%	0.52 [-0.34, 1.37]
Rodríguez - Rosell et al. 2021 squat AL (a)	113.3	25.2	16	99.2	22.9	16	6.4%	0.57 [-0.14, 1.28]
Rodríguez - Rosell et al. 2021 squat AL (b)	109.3	18.3	16	100.7	17.6	16	6.5%	0.47 [-0.24, 1.17]
Rodríguez-Rosell et al. 2020 squat AL (a)	116.6	20.7	12	100.8	24.6	12	4.7%	0.67 [-0.16, 1.50]
Rodríguez-Rosell et al. 2020 squat AL (b)	110.5	15.2	13	96.6	14.7	13	4.8%	0.90 [0.09, 1.71]
Rodríguez - Rosell et al. 2021 squat AL (a)	117.4	17.7	11	96.1	13.9	11	3.7%	1.29 [0.35, 2.22]
Rodríguez - Rosell et al. 2021 squat AL (b)	118.9	22.6	11	97.4	19.2	11	4.0%	0.99 [0.09, 1.88]
Rodríguez - Rosell et al. 2021 squat AL (c)	111.7	19.2	11	96.8	15.1	11	4.1%	0.83 [-0.05, 1.71]
**Subtotal (95% CI)**			**263**			**263**	**100.0%**	**0.76 [0.58, 0.94]**
Heterogeneity: Tau² = 0.00; Chi² = 6.92, df = 20 (P = 1.00); I² = 0%								
Test for overall effect: Z = 8.33 (P < 0.00001)								
**Total (95% CI)**			**263**			**263**	**100.0%**	**0.76 [0.58, 0.94]**
Heterogeneity: Tau² = 0.00; Chi² = 6.92, df = 20 (P = 1.00); I² = 0%								
Test for overall effect: Z = 8.33 (P < 0.00001)								
Test for subgroup differences: Not applicable								

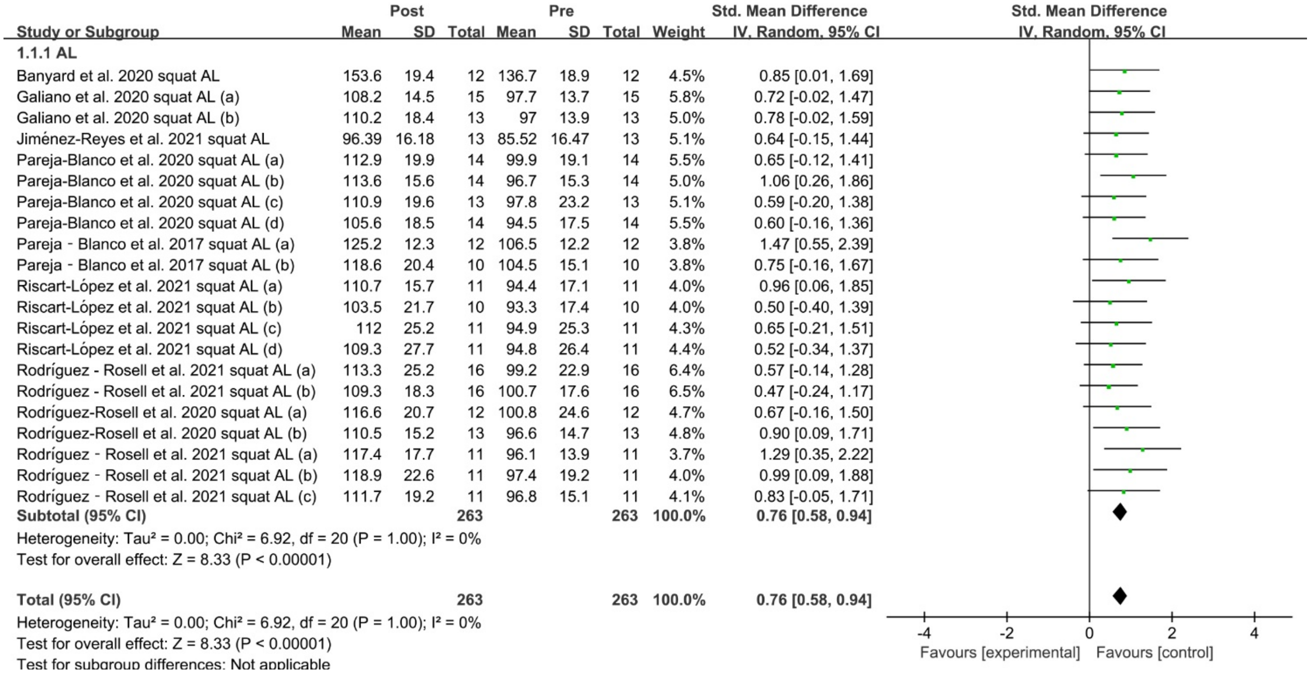

Figure S1. The effect of VBT on 1RM.

Study or Subgroup	Post Mean	Post SD	Post Total	Pre Mean	Pre SD	Pre Total	Weight	Std. Mean Difference IV, Random, 95% CI
**1.1.1 AL**								
Banyard et al. 2020 2020 T5 AL	1.09	0.06	12	1.16	0.06	12	3.9%	-1.13 [-2.00, -0.25]
Banyard et al. 2020 T10 AL	1.83	0.07	12	1.9	0.08	12	4.1%	-0.90 [-1.75, -0.05]
Banyard et al. 2020 T20 AL	3.14	0.12	12	3.2	0.11	12	4.5%	-0.50 [-1.32, 0.31]
Galiano et al. 2020 T20 AL (a)	2.94	0.13	15	3.09	0.15	15	5.0%	-1.04 [-1.81, -0.27]
Galiano et al. 2020 T20 AL (b)	2.96	0.12	13	3.07	0.16	13	4.6%	-0.75 [-1.55, 0.05]
Jiménez-Reyes et al. 2021 T10 AL	1.84	0.06	13	1.86	0.06	13	4.9%	-0.32 [-1.10, 0.45]
Jiménez-Reyes et al. 2021 T20 AL	3.14	0.08	13	3.17	0.08	13	4.9%	-0.36 [-1.14, 0.41]
Pareja - Blanco et al. 2017 T20 AL (a)	2.99	0.1	12	3	0.11	12	4.6%	-0.09 [-0.89, 0.71]
Pareja - Blanco et al. 2017 T20 AL (b)	3.02	0.08	10	2.99	0.09	10	3.8%	0.34 [-0.55, 1.22]
Riscart-López et al. 2021 T20 AL (a)	2.94	0.11	11	3	0.14	11	4.1%	-0.46 [-1.31, 0.39]
Riscart-López et al. 2021 T20 AL (b)	2.99	0.1	10	3.03	0.11	10	3.8%	-0.36 [-1.25, 0.52]
Riscart-López et al. 2021 T20 AL (c)	2.93	0.11	11	2.99	0.14	11	4.1%	-0.46 [-1.31, 0.39]
Riscart-López et al. 2021 T20 AL (d)	2.97	0.14	11	3.02	0.16	11	4.2%	-0.32 [-1.16, 0.52]
Rodríguez-Rosell et al. 2020 T10 AL (a)	1.76	0.08	12	1.79	0.07	12	4.5%	-0.39 [-1.19, 0.42]
Rodríguez-Rosell et al. 2020 T10 AL (b)	1.76	0.08	13	1.75	0.09	13	5.0%	0.11 [-0.66, 0.88]
Rodríguez-Rosell et al. 2020 T20 AL (a)	3.04	0.12	12	3.08	0.12	12	4.6%	-0.32 [-1.13, 0.48]
Rodríguez-Rosell et al. 2020 T20 AL (b)	3.07	0.14	13	3.06	0.15	13	5.0%	0.07 [-0.70, 0.84]
Rodríguez - Rosell et al. 2021 T10 AL (a)	1.71	0.06	11	1.77	0.07	11	3.8%	-0.89 [-1.77, -0.00]
Rodríguez - Rosell et al. 2021 T10 AL (b)	1.73	0.06	11	1.75	0.09	11	4.2%	-0.25 [-1.09, 0.59]
Rodríguez - Rosell et al. 2021 T10 AL (c)	1.75	0.09	11	1.75	0.12	11	4.2%	0.00 [-0.84, 0.84]
Rodríguez - Rosell et al. 2021 T20 AL (a)	2.99	0.08	11	3.06	0.1	11	3.9%	-0.74 [-1.61, 0.13]
Rodríguez - Rosell et al. 2021 T20 AL (b)	2.98	0.09	11	3.04	0.15	11	4.1%	-0.47 [-1.32, 0.38]
Rodríguez - Rosell et al. 2021 T20 AL (c)	3.03	0.13	11	3.05	0.13	11	4.2%	-0.15 [-0.99, 0.69]
**Subtotal (95% CI)**			**271**			**271**	**100.0%**	**-0.40 [-0.57, -0.23]**
Heterogeneity: Tau² = 0.00; Chi² = 17.12, df = 22 (P = 0.76); I² = 0%								
Test for overall effect: Z = 4.58 (P < 0.00001)								
**Total (95% CI)**			**271**			**271**	**100.0%**	**-0.40 [-0.57, -0.23]**
Heterogeneity: Tau² = 0.00; Chi² = 17.12, df = 22 (P = 0.76); I² = 0%								
Test for overall effect: Z = 4.58 (P < 0.00001)								
Test for subgroup differences: Not applicable								

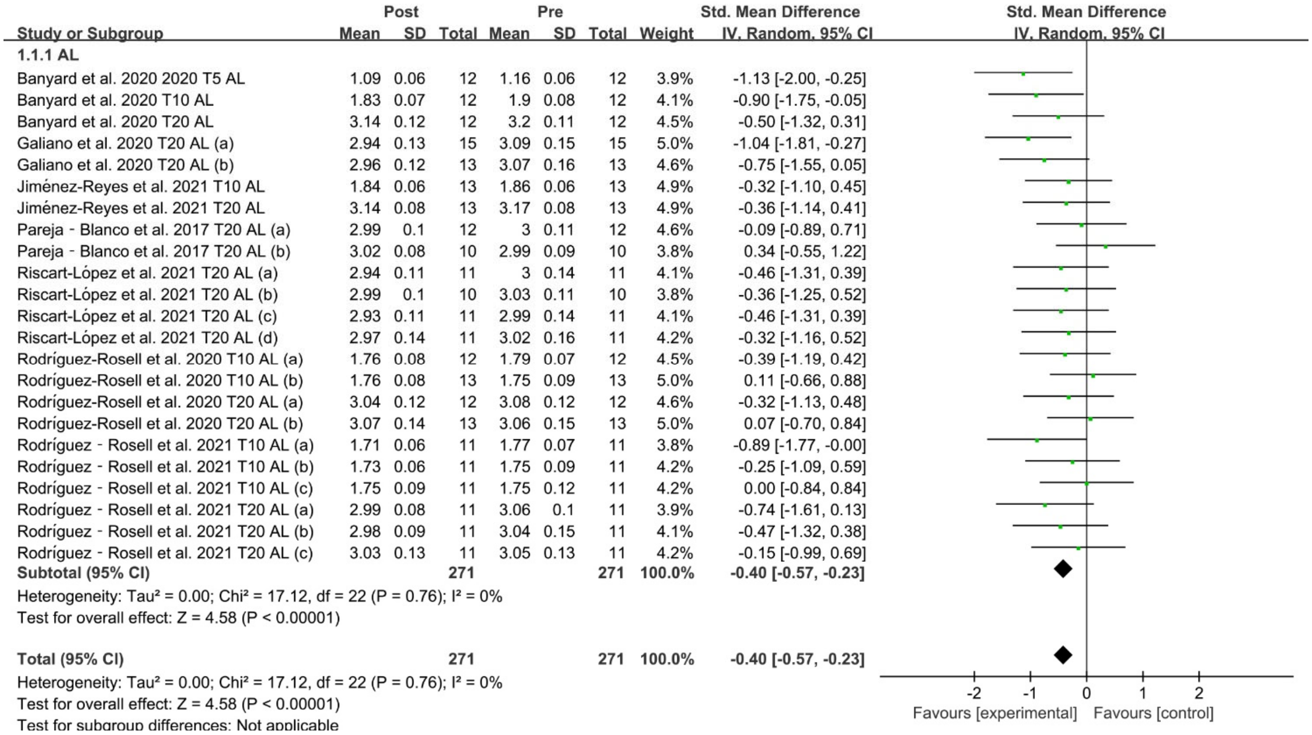

Figure S2. The effect of VBT on sprint time.

Study or Subgroup	Post Mean	Post SD	Post Total	Pre Mean	Pre SD	Pre Total	Weight	Std. Mean Difference IV, Random, 95% CI
**1.1.1 AL**								
Galiano et al. 2020 CMJ AL (a)	37.5	6.9	15	34.3	6.7	15	7.8%	0.46 [-0.27, 1.18]
Galiano et al. 2020 CMJ AL (b)	36.9	4.6	13	33.9	4.9	13	6.6%	0.61 [-0.18, 1.40]
Jiménez-Reyes et al. 2021 CMJ AL	40.31	3.71	13	37.35	3.47	13	6.3%	0.80 [-0.01, 1.60]
Pareja - Blanco et al. 2017 CMJ AL (a)	44.2	6	12	40.5	6	12	6.1%	0.60 [-0.23, 1.42]
Pareja - Blanco et al. 2017 CMJ AL (b)	42.5	5.8	10	41	4.3	10	5.3%	0.28 [-0.60, 1.16]
Riscart-López et al. 2021 CMJ AL (a)	40.3	4.7	11	38.3	5.2	11	5.7%	0.39 [-0.46, 1.23]
Riscart-López et al. 2021 CMJ AL (b)	40.2	5	10	37.2	5.2	10	5.1%	0.56 [-0.33, 1.46]
Riscart-López et al. 2021 CMJ AL (c)	43	8.7	11	38.8	7.2	11	5.7%	0.51 [-0.35, 1.36]
Riscart-López et al. 2021 CMJ AL (d)	39.9	7.1	11	37.2	7.5	11	5.8%	0.36 [-0.49, 1.20]
Rodríguez-Rosell et al. 2020 CMJ AL (a)	41.2	6.1	12	37.7	5.6	12	6.1%	0.58 [-0.24, 1.40]
Rodríguez-Rosell et al. 2020 CMJ AL (b)	40.6	6.6	13	38.4	5	13	6.8%	0.36 [-0.41, 1.14]
Rodríguez-Rosell et al. 2021 CMJ AL (a)	41.2	6	16	36.7	4.6	16	7.8%	0.82 [0.10, 1.55]
Rodríguez-Rosell et al. 2021 CMJ AL (b)	39.5	4.9	16	36.3	4.9	16	8.1%	0.64 [-0.08, 1.35]
Rodríguez - Rosell et al. 2021 CMJ AL (a)	43.8	6.6	11	39.1	5.7	11	5.4%	0.73 [-0.14, 1.60]
Rodríguez - Rosell et al. 2021 CMJ AL (b)	41.8	4.5	11	39.8	4.7	11	5.7%	0.42 [-0.43, 1.26]
Rodríguez - Rosell et al. 2021 CMJ AL (c)	40.8	6.4	11	39	6.3	11	5.8%	0.27 [-0.57, 1.11]
**Subtotal (95% CI)**			**196**			**196**	**100.0%**	**0.53 [0.33, 0.73]**
Heterogeneity: Tau² = 0.00; Chi² = 2.64, df = 15 (P = 1.00); I² = 0%								
Test for overall effect: Z = 5.15 (P < 0.00001)								
**Total (95% CI)**			**196**			**196**	**100.0%**	**0.53 [0.33, 0.73]**
Heterogeneity: Tau² = 0.00; Chi² = 2.64, df = 15 (P = 1.00); I² = 0%								
Test for overall effect: Z = 5.15 (P < 0.00001)								
Test for subgroup differences: Not applicable								

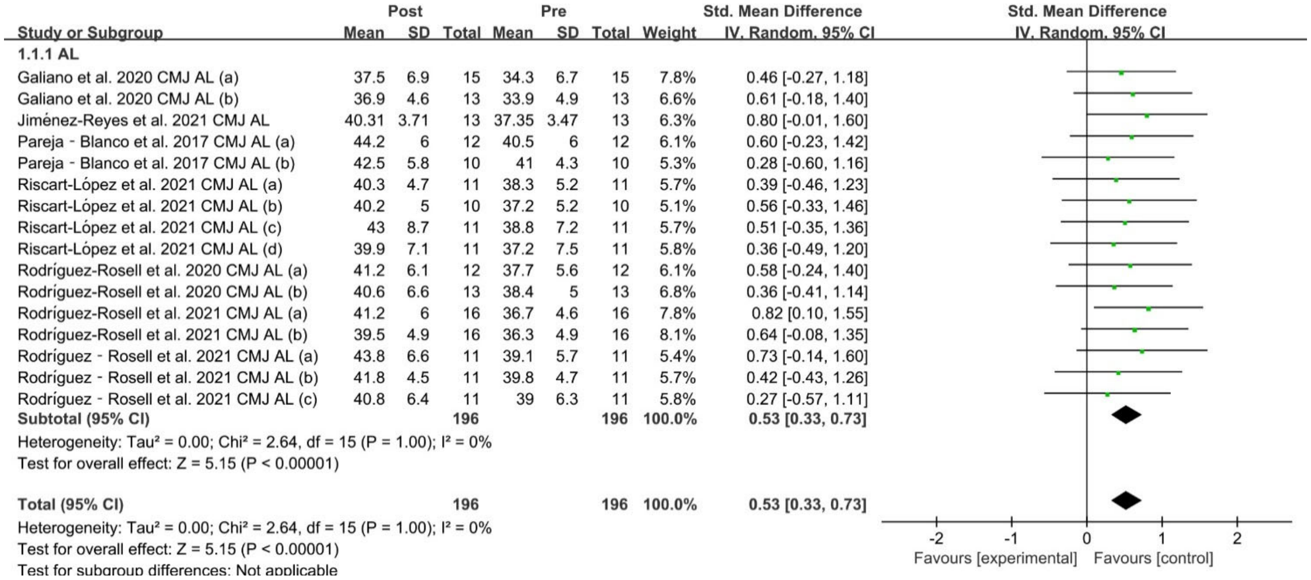

Figure S3. The effect of VBT on CMJ.

Study or Subgroup	Post Mean	Post SD	Post Total	Pre Mean	Pre SD	Pre Total	Weight	Std. Mean Difference IV, Random, 95% CI
**1.1.1 AL**								
Pareja-Blanco et al. 2020 squat AL (a)	14.2	6	14	9.2	2.5	14	10.2%	1.06 [0.26, 1.85]
Pareja-Blanco et al. 2020 squat AL (b)	19.7	7.9	14	10	3.2	14	8.8%	1.56 [0.70, 2.42]
Pareja-Blanco et al. 2020 squat AL (c)	17.1	7.2	13	8.4	2.3	13	8.1%	1.58 [0.68, 2.47]
Pareja-Blanco et al. 2020 squat AL (d)	16.1	7.5	14	8.4	2.8	14	9.5%	1.32 [0.49, 2.15]
Rodríguez - Rosell et al. 2021 squat AL (a)	23.4	9.7	16	15.1	6.4	16	11.9%	0.98 [0.25, 1.72]
Rodríguez - Rosell et al. 2021 squat AL (b)	20.7	8	16	17.5	8.2	16	13.2%	0.39 [-0.32, 1.09]
Rodríguez-Rosell et al. 2020 squat AL (a)	19.8	5	12	11.8	3.1	12	6.8%	1.86 [0.87, 2.84]
Rodríguez-Rosell et al. 2020 squat AL (b)	22.08	4.7	13	13.9	6	13	8.4%	1.47 [0.59, 2.35]
Rodríguez - Rosell et al. 2021 squat AL (a)	17.6	4.2	11	11.5	3.5	11	7.0%	1.52 [0.55, 2.49]
Rodríguez - Rosell et al. 2021 squat AL (b)	17.6	4.2	11	12	4.3	11	7.6%	1.27 [0.34, 2.20]
Rodríguez - Rosell et al. 2021 squat AL (c)	18.3	6.3	11	12.9	6	11	8.5%	0.84 [-0.04, 1.72]
**Subtotal (95% CI)**			**145**			**145**	**100.0%**	**1.19 [0.93, 1.45]**
Heterogeneity: Tau² = 0.00; Chi² = 10.21, df = 10 (P = 0.42); I² = 2%								
Test for overall effect: Z = 9.02 (P < 0.00001)								
**Total (95% CI)**			**145**			**145**	**100.0%**	**1.19 [0.93, 1.45]**
Heterogeneity: Tau² = 0.00; Chi² = 10.21, df = 10 (P = 0.42); I² = 2%								
Test for overall effect: Z = 9.02 (P < 0.00001)								
Test for subgroup differences: Not applicable								

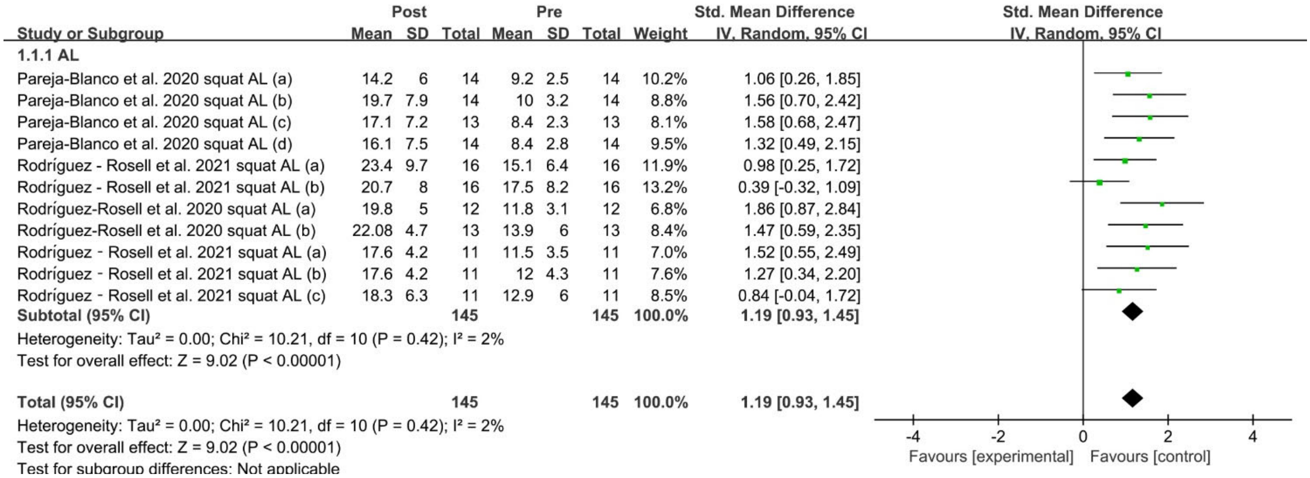

Figure S4. The effect of VBT on MNR.
